# Comparison of developmental trauma between immigrant and non-immigrant psychotic patients

**DOI:** 10.1192/j.eurpsy.2021.1943

**Published:** 2021-08-13

**Authors:** A. Trabsa, A. Llimona, L. Vargas, F. Casanovas, M. Martín, A. Valiente, A. Moreno, B. Amann, V. Pérez-Solà

**Affiliations:** 1 Psychiatry And Legal Medicine, Autonomous University of Barcelona, Barcelona, Spain; 2 Inad-parc De Salut Mar, Hospital del Mar, Barcelona, Spain; 3 Institut De Neuropsiquiatria I Addiccions (inad), Hospital del Mar, Barcelona, Spain

**Keywords:** transcultural psychiatry, trauma, psychosis, migration psychiatry

## Abstract

**Introduction:**

Meta-analytic evidence suggests that migrants have higher risk for psychotic disorders. Likewise, growing evidence relate developmental trauma (emotional, sexual, physical abuse and neglect in childhood or adolescence) as a causal factor for psychotic symptoms. However, few studies examine developmental trauma in migrant populations.

**Objectives:**

The aim of this study is to describe and compare developmental trauma exposure prevalence between immigrant and non-immigrant psychotic patients in Barcelona.

**Methods:**

Patients who have presented, according DSM-V criteria, one or more non-affective psychotic episodes, were recruited in Acute and Chronic inpatients units at Hospital del Mar (Barcelona), leading to a total sample of 77 patients. Demographic characteristics of patients, clinical data and main pharmacological treatment were recorded through a questionnaire. Developmental trauma exposure was assessed by Childhood Trauma Questionnaire (CTQ). Comparative analysis was performed with IBM SPSS using Chi-Square Test and t-Student test.

**Results:**

From a total of 77 patients, 43 were immigrants and 34 were non-immigrants. Exposure to traumatic events showed significant differences between immigrants and non-immigrant in Child emotional abuse (64,4% immigrants, 35,3% non-immigrant), Child physical abuse (51,2% immigrants, 14,7% non-immigrant), Child Sexual Abuse (41,9% immigrants, 11,8% non-immigrant) and physical neglect (62,8% immigrants, 26,5% non-immigrant). Emotional neglect exposure was no significant between both groups. Total mean CTQ score was 37,53 in immigrants group and 52,60 in non-immigrant group.

**Conclusions:**

According to our results, there are important and significant differences in developmental trauma exposure between immigrant and non-immigrant psychotic patients. These results should be considered by clinicians in order to design assessment program for this population.

**Disclosure:**

No significant relationships.

